# Circulating microRNA profiles based on direct S‐Poly(T)Plus assay for detection of coronary heart disease

**DOI:** 10.1111/jcmm.15001

**Published:** 2020-04-28

**Authors:** Mingyang Su, Yanqin Niu, Quanjin Dang, Junle Qu, Daling Zhu, Zhongren Tang, Deming Gou

**Affiliations:** ^1^ Shenzhen Key Laboratory of Microbial Genetic Engineering Vascular Disease Research Center College of Life Sciences and Oceanography Guangdong Provincial Key Laboratory of Regional Immunity and Diseases Carson International Cancer Center Shenzhen University Shenzhen Guangdong China; ^2^ Key Laboratory of Optoelectronic Devices and Systems of Ministry of Education and Guangdong Province College of Optoelectronic Engineering Shenzhen University Shenzhen Guangdong China; ^3^ Department of Biopharmaceutical Sciences College of Pharmacy Harbin Medical University Harbin Heilongjiang China; ^4^ Third Cardiovascular Department Mudanjiang City Second People's Hospital Mudanjiang Heilongjiang China

**Keywords:** biomarker, circulating miRNA, coronary heart disease (CHD), direct S‐Poly(T)Plus, reverse transcription quantitative polymerase chain reaction (RT‐qPCR)

## Abstract

Coronary heart disease (CHD) is one of the leading causes of heart‐associated deaths worldwide. Conventional diagnostic techniques are ineffective and insufficient to diagnose CHD with higher accuracy. To use the circulating microRNAs (miRNAs) as non‐invasive, specific and sensitive biomarkers for diagnosing of CHD, 203 patients with CHD and 144 age‐matched controls (126 high‐risk controls and 18 healthy volunteers) were enrolled in this study. The direct S‐Poly(T)Plus method was used to identify novel miRNAs expression profile of CHD patients and to evaluate their clinical diagnostic value. This method is an RNA extraction‐free and robust quantification method, which simplifies procedures, reduces variations, in particular increases the accuracy. Twelve differentially expressed miRNAs between CHD patients and high‐risk controls were selected, and their performances were evaluated in validation set‐1 with 96 plasma samples. Finally, six (miR‐15b‐5p, miR‐29c‐3p, miR‐199a‐3p, miR‐320e, miR‐361‐5p and miR‐378b) of these 12 miRNAs were verified in validation set‐2 with a sensitivity of 92.8% and a specificity of 89.5%, and the AUC was 0.971 (95% confidence interval, 0.948‐0.993, *P* < .001) in a large cohort for CHD patients diagnosis. Plasma fractionation indicated that only a small amount of miRNAs were assembled into EVs. Direct S‐Poly(T)Plus method could be used for disease diagnosis and 12 unique miRNAs could be used for diagnosis of CHD.

## 1 INTRODUCTION

Coronary heart disease (CHD) is a life‐threatening disease and remains a leading cause of heart‐associated deaths for adults worldwide, which develops over time due to genetic and environment factors with complex pathology.^1^ Early diagnosis, effective prevention and therapy for CHD pose a major challenge to the entire medical community.^2^ Without the help of well‐established invasive coronary angiogram (CAG) techniques, CHD is hard to diagnose. CAG is difficult to perform if multiple vessels are affected or the artery is narrowed at multiple locations. On the other hand, CAG may not be effective against very hard atherosclerotic plaques.^3^ In recent years, application of non‐invasive molecular biomarkers is emerging as a powerful approach to diagnosis and prediction of CHD, and circulating microRNA (miRNA) biomarkers have shown great potential for clinical diagnosis of CHD, particularly for the early diagnosis and prognosis of CHD.^4^

microRNAs are endogenous small non‐coding RNAs consisting of 21‐25 nucleotides in length and function as key mediators of RNA silencing and post‐transcriptional regulation of gene expression.^5^ Recent evidences suggest miRNAs are involved in cardiac regeneration,^6^ remodelling^7^ and hypertrophy^8^ along with their involvement in cardiac development. Circulating miRNAs are released by cells,^9^ secreted by membrane‐bound vesicles^10^ or exported by protein‐protected miRNA complexes,^11^ exhibiting remarkable stability and resistance to RNase activity. Emerging evidences suggest that different combinations of plasma miRNAs can be used to identify various human diseases,^12^ attracting considerable interest in using circulating miRNAs as biomarkers. With the hypothesis that muscle‐ or heart‐specific miRNAs are released into circulation from the injured heart,^13^ circulating miRNAs demonstrate significant dynamic change in human serum and plasma. To date, non‐invasive and blood‐based studies have examined miRNA expression profiles to identify novel miRNA biomarkers for CHD diagnosis. Although these findings suggest that some circulating miRNAs might be potential diagnosis markers, most of the results are based on a limited number of patients and few specific miRNAs.^14^, ^15^ And almost no related studies could be used as an auxiliary technique for CHD prevention, prediction, diagnosis and the effectiveness of therapies, due to their time‐consuming operation and imprecise quantification.

In order to promote clinical application of circulating miRNAs as biomarkers, an accurate, convenient and inexpensive profiling approach is needed. By combining S‐Poly(T)Plus method and extraction‐free miRNAs isolation technique, we precisely quantify miRNAs expression in 1 hour, making this method effective in monitoring CHD progression. More importantly, longitudinal measurements of miRNAs in CHD patients may provide further insight into individual temporal patterns and the patient's ensuing risk for disease progression and adverse outcome.^16^ Here, using plasmas obtained from CHD patients and high‐risk individuals, we unearthed a group of miRNAs that can serve as non‐invasive biomarkers for the diagnosis of CHD, and we evaluated their performances. This quick quantification method has a great potential to be used in clinical investigation.

## 2 MATERIALS AND METHODS

### 2.1 Study population and ethics statement

A total of 203 consecutive CHD patients were recruited in this study in cardiology department, Mudanjiang City Second People's Hospital (Heilongjiang, China) between April 2016 and August 2018. CHD diagnosis was confirmed by coronary angiography and defined as angiographic evidence of more than 50% luminal narrowing in at least one segment of a main epicardial coronary artery.^17^ A total of 126 high‐risk controls were recruited and the criterion for the high‐risk cohort was defined as the individuals having chest pains, fatigue but without obvious lumen diameter stenosis confirmed by coronary angiography, blood test and fully body examination. The patients with clinical diagnosis of acute myocardial infarction were excluded. Eighteen healthy volunteers who undertook a routine physical examination were included as healthy volunteers. The clinicopathologic and histologic information about patients and high‐risk controls was obtained from the medical and pathological records in hospital. The characteristics of patients, high‐risk controls and healthy volunteers enrolled in this study are given in Tables 1, 2, 3. The present study was approved by the ethics committee board of Mudanjiang City Second People's Hospital. All the CHD patients, high‐risk controls and healthy volunteers signed an informed consent document.

**Table 1:** Demographical and clinical features of coronary heart disease (CHD) patients and high‐risk controls in the discovery set and training set

	Discovery set			Training set		
	High‐risk contro	CHD	*P* value	High‐risk control	CHD	*P* value
	3 Pools (n = 18)	3 Pools (n = 18)		INDV (n = 18)	INDV (n = 24)	
Age (y)	51.11 ± 11.85	56.56 ± 8.91	.31	56.65 ± 8.36	64.69 ± 7.54	.001
Female	13	11		12	13	
Male	5	7		6	11	
Clinical features						
Height (cm)	165.00 ± 5.77	167.00 ± 7.33	.29	164.04 ± 5.50	168.22 ± 8.03	.14
Body weight (kg)	63.80 ± 7.75	67.20 ± 9.49	.26	61.58 ± 4.86	69.37 ± 8.58	.021
Body mass index	23.40 ± 1.57	24 ± 2.4	.41	22.88 ± 1.43	24.47 ± 1.95	.02
Blood parameter						
TCH (mmol/L)	5.11 ± 1.11	5.33 ± 0.95	.55	5.21 ± 1.07	5.31 ± 1.62	.84
TG (mmol/L)	1.66 ± 1.41	1.51 ± 1.044	.73	1.37 ± 1.09	2.09 ± 1.68	.09
HDL (mmol/L)	1.56 ± 0.53	1.60 ± 0.60	.85	1.46 ± 0.61	2.17 ± 1.34	.06
LDL (mmol/L)	2.99 ± 1.07	3.18 ± 0.91	.59	2.77 ± 1.06	2.35 ± 1.29	.22
Hcy (μmol/L)	9.06 ± 5.38	9.81 ± 5.13	.68	11.77 ± 5.61	11.12 ± 6.41	.79
UA (μmol/L)	302.53 ± 122.95	338 ± 115.72	.36	291.60 ± 170.00	353.28 ± 126.30	.32
Urea (mmol/L)	5.43 ± 1.45	4.85 ± 1.52	.32	4.98 ± 1.31	5.72 ± 1.70	.15
Creatinine (μmol/L)	54.87 ± 9.57	54.23 ± 11.67	.88	56.95 ± 14.74	57.40 ± 12.91	.27
Apolipoprotein A (g/L)	1.37 ± 0.32	1.45 ± 0.19	.47	1.35 ± 0.28	1.29 ± 0.15	.38
Apolipoprotein B (g/L)	0.95 ± 0.35	0.98 ± 0.30	.81	0.98 ± 0.35	1.03 ± 0.34	.72
Lipoprotein(a) (mg/L)	156.29 ± 132.94	160.99 ± 173.45	.94	111.7 ± 94.2	140.32 ± 113.84	.46
White blood cell (10^9^/L)	6.52 ± 1.93	7.76 ± 2.86	.17	6.18 ± 1.80	7.66 ± 1.69	.03
Lymphocyte (%)	31.20 ± 7.42	25.31 ± 10.48	.09	32.80 ± 9.32	26.04 ± 5.99	.04
Neutrophile granulocyte (%)	59.89 ± 7.35	66.65 ± 11.997	.07	57.67 ± 10.54	64.52 ± 6.69	.06
Red blood cell (10^12^/L)	4.37 ± 0.61	4.86 ± 0.69	.04	4.38 ± 0.58	4.41 ± 0.52	.91
Hemoglobin (g/L)	199 ± 244.66	150.53 ± 20.29	.44	137.27 ± 17.43	129.78 ± 32.79	.37
Hematocrit (%)	39.41 ± 5.13	43.33 ± 5.07	.04	78.49 ± 127.87	40.18 ± 4.39	.17
Platelet (10^9^/L)	231.38 ± 68.45	245 ± 54.26	.54	214.50 ± 49.00	244.11 ± 54.92	.09
Other disease						
Diabetes mellitus, n (%)	0 (0)	12 (66.67)		0 (0)	10 (41.67)	
Hypertension, n (%)	2 (11.11)	4 (22.22)		6 (33.33)	18 (75.00)	
Cerebral vascular event, n (%)	0 (0)	2 (11.11)		0 (0)	0 (0)	
Smoking status, n(%) Angiocardiography results	1 (5.56)	5 (27.78)		1 (4.35)	9 (32.14)	
LM	0	1		0	3	
LAD	2 (slight)	17		12 (slightly)	23	
LCX	1	7		6	22	
D	0	3		0	1	
OM	0	0		0	0	
RCA	1 (slight)	10		4 (slightly)	21	
PD	0	0		0	0	
PL	0	0		0	0	

Note

Data are expressed as the mean ± SD or as n (%). Body mass index (BMI) was calculated as weight divided by height squared. Student's t test was used to calculate *P* value.

Abbreviations: D, diagonal branch; Hcy, homocysteine; HDL, high‐density lipoprotein; INDV, individual; LAD, left anterior descending; LCX, left circumflex branch; LDL, low‐density lipoprotein; LM, left main coronary artery; OM, obtuse marginal branch; PD, posterior descending branch; PL, posterior branch of left ventricle; RCA, right coronary artery; TCH, total cholesterol; TG, triglycerides; UA, uric acid.

**Table 2:** Demographical and clinical features of coronary heart disease (CHD) patients and high‐risk controls in validation set‐1 and validation set‐2

	Validation set‐1			Validation set‐2		
	High‐risk control	CHD	*P* value	High‐risk control	CHD	*P* value
	INDV (n = 48)	INDV (n = 48)		INDV (n = 60)	INDV (n = 95)	
Age (y)	53.92 ± 9.02	60.92 ± 12.56	.15	165.53 ± 6.48	168.05 ± 44.83	.27
Female	31	21	.18	65.02 ± 7.19	68.05 ± 19.76	.24
Male	17	27	.65	23.71 ± 1.97	24.07 ± 2.47	.33
Clinical features						
Height (cm)	165.69 ± 6.68	167.87 ± 34.69	.15	165.53 ± 6.48	168.05 ± 44.83	.27
Body weight (kg)	66.11 ± 8.19	68.50 ± 16.20	.18	65.02 ± 7.19	68.05 ± 19.76	.24
Body mass index	23.05 ± 2.25	24.27 ± 2.33	.65	23.71 ± 1.97	24.07 ± 2.47	.33
Blood parameter						
TCH (mmol/L)	4.71 ± 1.46	4.95 ± 1.72	.36	4.81 ± 1.44	4.78 ± 1.46	.89
TG (mmol/L)	1.61 ± 1.17	2.04 ± 1.56	.15	1.583 ± 1.19	1.64 ± 0.99	.76
HDL (mmol/L)	1.31 ± 0.49	1.34 ± 0.49	.66	1.34 ± 0.53	1.33 ± 0.71	.91
LDL (mmol/L)	3.04 ± 1.15	2.96 ± 1.19	.77	2.94 ± 1.14	3.09 ± 1.11	.39
Hcy (μmol/L)	8.94 ± 5.02	10.75 ± 6.52	.16	9.36 ± 5.29	10.75 ± 6.16	.18
UA (μmol/L)	295.71 ± 134.57	340.88 ± 144.36	.14	290.73 ± 148.27	343.33 ± 153.03	.03
Urea (mmol/L)	5.32 ± 1.24	5.52 ± 1.52	.5	5.23 ± 1.22	5.58 ± 1.69	.19
Creatinine (μmol/L)	65.65 ± 13.64	62.10 ± 14.62	.26	62.89 ± 13.12	66.39 ± 17.98	.21
Apolipoprotein A (g/L)	1.34 ± 0.22	1.45 ± 0.19	.02	1.34 ± 0.25	1.27 ± 0.16	.09
Apolipoprotein B (g/L)	0.86 ± 0.24	0.90 ± 0.29	.47	0.89 ± 0.29	5.71 ± 41.93	.32
Lipoprotein(a) (mg/L)	146.90 ± 130.43	197.79 ± 216.11	.24	139.61 ± 127.29	198.89 ± 215.23	.06
White blood cell (10^9^/L)	6.60 ± 1.73	6.76 ± 1.51	.55	6.56 ± 1.77	6.67 ± 2.12	.72
Lymphocyte (%)	28.99 ± 7.59	27.32 ± 9.38	.35	29.45 ± 8.04	32.52 ± 27.05	.32
Neutrophil granulocyte (%)	61.72 ± 8.88	64.12 ± 11.11	.26	61.41 ± 9.17	59.92 ± 10.11	.36
Red blood cell (10^12^/L)	4.62 ± 0.62	4.76 ± 0.69	.36	4.58 ± 0.61	4.68 ± 0.51	.34
Hemoglobin (g/L)	167.78 ± 144.88	151.54 ± 15.87	.45	161.79 ± 130.42	146.23 ± 14.94	.38
Hematocrit (%)	41.62 ± 5.35	43.78 ± 4.074	.04	48.81 ± 57.99	42.36 ± 3.98	.41
Platelet (10^9^/L)	241.98 ± 68.48	216.671 ± 50.41	.08	237.67 ± 66.56	220.59 ± 44.74	.11
Other disease						
Diabetes mellitus, n (%)	1 (2.08)	7 (14.58)		1 (1.67)	14 (14.74)	
Hypertension, n (%)	12 (25.00)	18 (37.50)		15 (25.00)	43 (45.26)	
Cerebral vascular event, n (%)	0 (0)	1 (2.08)		0 (0)	1 (1.05)	
Smoking status, n(%) Angiocardiography results	7 (14.58)	13 (27.08)		6 (10.00)	27 (28.42)	
LM	1 (slight)	4		1	7	
LAD	14 (slight)	44		22 (slight)	84	
LCX	1 (slight)	32		5	64	
D	0	5		0	11	
OM	0	2		0	4	
RCA	1 (slight)	42		5 (slight)	76	
PD	0	0		0	5	
PL	0	0		0	3	

Abbreviations: D, diagonal branch; Hcy, homocysteine; HDL, high‐density lipoprotein; INDV, individual; LAD, left anterior descending; LCX, left circumflex branch; LDL, low‐density lipoprotein; LM, left main coronary artery; OM, obtuse marginal branch; PD, posterior descending branch; PL, posterior branch of left ventricle; RCA, right coronary artery; TCH, total cholesterol; TG, triglycerides; UA, uric acid.

**Table 3:** Demographical and clinical features of coronary heart disease (CHD) patients and healthy volunteers in evaluation set

	Evaluation set		
	Healthy volunteers	CHD	*P* value
	INDV (n = 18)	INDV (n = 18)	
Age (y)	53.92 ± 9.02	60.92 ± 12.56	.06
Female	10	9	
Male	8	9	
Clinical features			
Height (cm)	170.69 ± 6.68	167.64 ± 40.03	.25
Body weight (kg)	64.11 ± 8.19	68.88 ± 21.84	.38
Body mass index	22.05 ± 1.25	24.45 ± 4.77	75
Blood parameter			
TCH (mmol/L)	—	4.83 ± 1.63	—
TG (mmol/L)	—	1.24 ± 0.88	—
HDL (mmol/L)	—	1.47 ± 0.51	—
LDL (mmol/L)	—	2.99 ± 1.30	—
Hcy (μmol/L)	—	1035 ± 7.42	—
UA (μmol/L)	—	330.13 ± 153.74	—
Urea (mmol/L)	—	5.57 ± 1.60	—
Creatinine (μmol/L)	—	61.94 ± 17.31	—
Apolipoprotein A (g/L)	—	1.40 ± 0.15	—
Apolipoprotein B (g/L)	—	0.91 ± 0.32	—
Lipoprotein(a) (mg/L)	—	271.12 ± 237.30	—
White blood cell (10^9^/L)	—	5.51 ± 1.27	—
Lymphocyte (%)	—	32.41 ± 7.71	—
Neutrophile granulocyte (%)	—	57.16 ± 8.14	—
Red blood cell (10^12^/L)	—	4.38 ± 0.54	—
Hemoglobin (g/L)	—	136.41 ± 18.44	—
Hematocrit (%)	—	39.85 ± 4.81	—
Platelet (10^9^/L)	—	222.65 ± 57.31	—
Other disease			
Diabetes mellitus, n (%)	0 (0)	1 (5.56)	
Hypertension, n (%)	2 (11.11)	5 (27.78)	
Cerebral vascular event, n (%)	0 (0)	0 (0)	
Smoking status, n(%) Angiocardiography results			
	0 (0)	3 (16.67)	
LM	0	2	
LAD	0	15	
LCX	0	8	
D	0	0	
OM	0	0	
RCA	0	15	
PD	0	0	
PL	0	0	

Abbreviations: D, diagonal branch; Hcy, homocysteine; HDL, high‐density lipoprotein; INDV, individual; LAD, left anterior descending; LCX, left circumflex branch; LDL, low‐density lipoprotein; LM, left main coronary artery; OM, obtuse marginal branch; PD, posterior descending branch; PL, posterior branch of left ventricle; RCA, right coronary artery; TCH, total cholesterol; TG, triglycerides; UA, uric acid.

### 2.2 Samples collection and processing

A total of 5 mL of venous blood was obtained into ethylenediaminetetraacetic acid (EDTA)‐containing tubes (BD, USA) from donors after overnight fasting. Samples were centrifuged at 1600 g for 10 minutes at 4°C to remove blood cells, followed by centrifugation at 16 000 g for 10 minutes at 4°C to completely remove cell debris.^18^ To guarantee the quality of samples, the haemolytic plasma which appeared pale red or pink was excluded from consideration. Plasma was collected and stored in aliquots into RNase/DNase‐free tubes at −80°C until analysis.

### 2.3 miRNAs profiling

**Figure 1:**
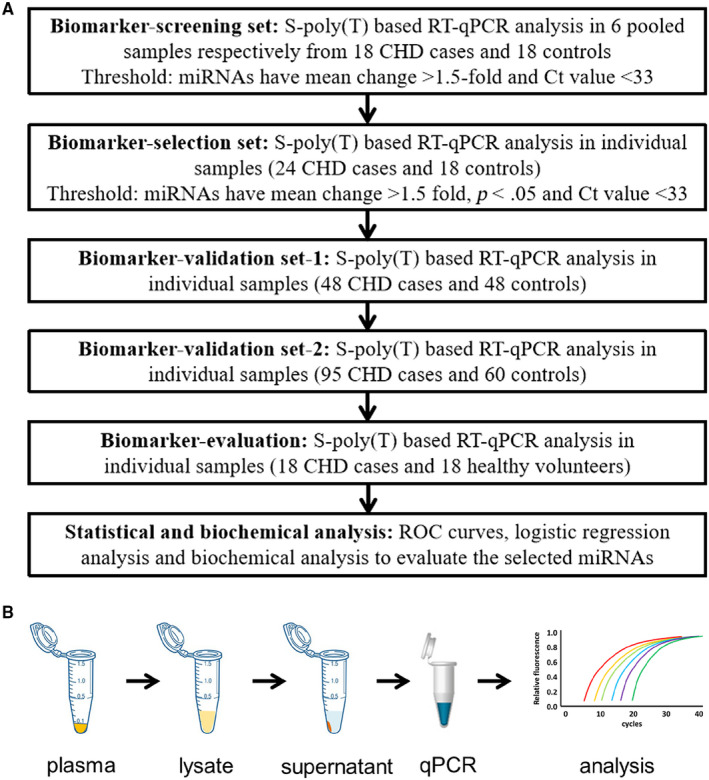
Study flow diagram. A, Experiment design illustrates the major steps of miRNAs screening as non‐invasive biomarker for CHD. All patients were enrolled at Mudanjiang City Second People's Hospital. B, Major steps of direct S‐Poly(T)Plus assay. CHD, coronary heart disease; RT‐qPCR, reverse transcription quantitative polymerase chain reaction

To identify potential biomarkers, we profiled miRNAs from pooled plasma and individual plasma. The whole study flow chat is shown in Figure 1. Firstly, we prepared three pools of 18 CHD patients and three pools of 18 high‐risk controls. Quantitative global profiling of plasma miRNAs was performed using the direct S‐Poly(T)Plus approach^19^ to screen from each pool (Files S1 and S2), and comparing the level of each miRNA in CHD and high‐risk groups. Secondly, the candidate miRNAs were detected in high‐risk individuals and CHD patients in two big cohorts of individual samples, respectively. Ultimately, selected miRNAs were evaluated in plasma from CHD patients and healthy volunteers.

### 2.4 Extraction‐free miRNAs isolation and quick quantification

Plasma for miRNAs detection was treated based on our optimized direct extraction method. Briefly, 20 μL thawed plasma was mixed thoroughly with 20 μL 2 × lysis buffer and 1 μL protease K, followed by incubation for 20 minutes at 50°C, 5 minutes at 95°C to denature protease K completely. The jelly products were centrifuged at 14 000 g for 5 minutes at 4°C to remove precipitants. The supernatant was preceded immediately for RT reaction.

Quantification was performed through S‐Poly(T)Plus method as described before. The level of miRNAs was calculated using 2^−ΔCt^ and normalized to global mean Ct value. Exogenous spike‐in cel‐miR‐54 was measured to evaluate the stability and to normalize candidate miNRAs. All sequences of miRNAs in this study were downloaded from miRBase 22.^20^ TaqMan probe and miRNA‐specific primer sequences (File S1) were designed in the laboratory and synthesized by IDT (Integrated DNA Technologies) and GENEWIZ. Candidate miRNAs were further validated by Sanger Sequencing.

### 2.5 Extracellular vesicles isolation, verification and miRNAs expression profiling

Extracellular vesicles (EVs) were isolated from plasma with differential centrifugation/ultracentrifugation (Figure 5A). About 1 mL plasma was diluted to 20 mL with ice‐cold PBS. The diluted plasma was centrifuged at 300 g for 10 minutes at 4°C to remove cell debris, followed by centrifugation at 10 000 g for 40 minutes at 4°C. The pellet was suspended in ice‐cold PBS and collected as big/middle size vesicles. Meanwhile, the 20 mL supernatant was centrifuged at 100 000 g in 70Ti ultracentrifuge rotor (Beckman Coulter) for 90 minutes at 4°C.^21^ The supernatant was recovered, and the pellet was suspended in 100 μL ice‐cold PBS. The suspended pellets were dissolved in RIPA Lysis and Extraction Buffer (Thermo Fisher). SDS‐PAGE was performed to separate proteins and then was subjected to immunoblot analysis. Antibodies were used to probe calnexin (Thermo Fisher), TSG101 (Abcam), CD63 (Abcam), CD9 (Abcam) and CD81 (Abcam). Blots were scanned using a Tanon 5200 (Tanon) imaging system. The supernatant and the suspended pellets were treated with RNAiso‐plus (TAKARA) to isolate miRNAs.^19^ microRNAs were analysed using RT‐qPCR as described above.

### 2.6 Statistical analyses

Statistical analyses were performed with GraphPad Prism version 7.0 (GraphPad Software, Inc), SPSS (version 21; IBM SPSS Statistics for Windows) and R (v3.4.4). The data were presented as the mean ± SEM for miRNA levels or mean ± SD for other variables. Non‐parametric Mann‐Whitney tests were used to compare miRNA levels between the CHD groups and high‐risk groups in discovery set. Student's *t* test was used to compare the differences in other variables between the two groups. *P* < .05 was considered statistically significant.

## 3 RESULTS

### 3.1 Baseline clinical characteristics of the study population

We recruited 347 participants including 203 CHD patients, 126 high‐risk controls and 18 healthy volunteers. All the CHD patients were selected on the basis of clinical parameters (eg chest pain and palpitation, history and laboratory value) combined with angiographic documentation (Figure S1). High‐risk controls were recruited from a large pool of individuals seeking a routine chest examination without obvious cardiovascular obstruction. High‐risk control subjects were matched to the patients by age and sex. The demographics and clinical features of the patients, high‐risk controls and healthy volunteers enrolled in this study are listed in Tables 1, 2, 3.

### 3.2 Identification of candidate miRNAs in discovery set

**Figure 2:**
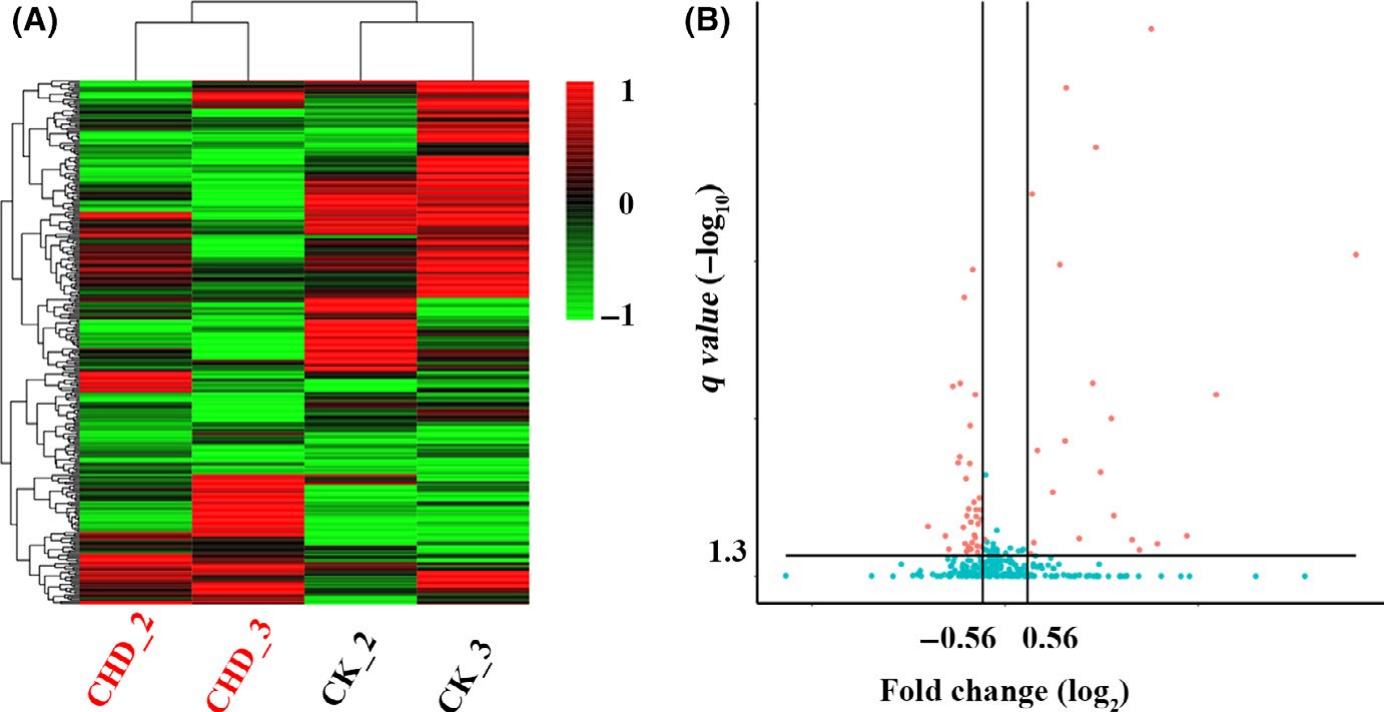
Profiling of 343 miRNAs in plasma of CHD patients and control individuals. A, Heatmap showing differentially expressed genome‐wide miRNA from plasma in high‐risk controls compared to CHD patients; red represents up‐regulated miRNAs and green represents down‐regulated miRNAs; CK stands for high‐risk control; CHD stands for CHD patient. B, Volcano plot showing the expression level of each miRNA in plasma with fold change (log_2_ ratio) against the confidence (−log_10_ adjusted *P* value); red dots represent the fold change >1.5, *P* < .05. Data are presented as relative expression (2^−ΔCt^), and expression is normalized to the global mean Ct value; the stability is evaluated by detecting spiked‐in (cel‐miR‐54). CHD, coronary heart disease

To identify novel miRNAs biomarkers for CHD diagnosis, we collected plasma from CHD patients and high‐risk controls. Firstly, we performed S‐Poly(T)Plus analysis to screen candidate miRNAs that showed obvious alteration in three paired plasma samples between CHD patients and high‐risk controls (Figure 1A,B). These 18 high‐risk controls which were selected in discovery set were reused in discovery, training and validation steps. As is shown in Table 1, there were no significant differences in the distribution of smoking, alcohol consumption, age and sex between these two groups. We compared the miRNA quick quantification method with conventional TRIzol isolation method, and the results indicated that quick quantification method could accurately and sensitively quantify circulating miRNA in plasma (Figure S2A‐C). Among 343 miRNAs scanned, 335 miRNAs were detected (Figure 2). In order to evaluate quality consistency of pooled plasma, we performed principal component analysis and excluded discrete samples from further analysis (Figure S2D). The miRNAs were considered to be regulated between these two groups based on following parameters: (a) Ct values < 33; (b) miRNAs showed >1.5‐fold change in relative expression; and (c) non‐parametric Kolmogorov‐Smirnov test *q* value <0.05. These criteria yielded a list of 59 differentially expressed miRNAs, 22 of which were up‐regulated and 37 down‐regulated in CHD patients compared with high‐risk controls (Figure 2; Figure S2D; File S2).

### 3.3 Confirmation of increased plasma miRNAs in training set

**Figure 3:**
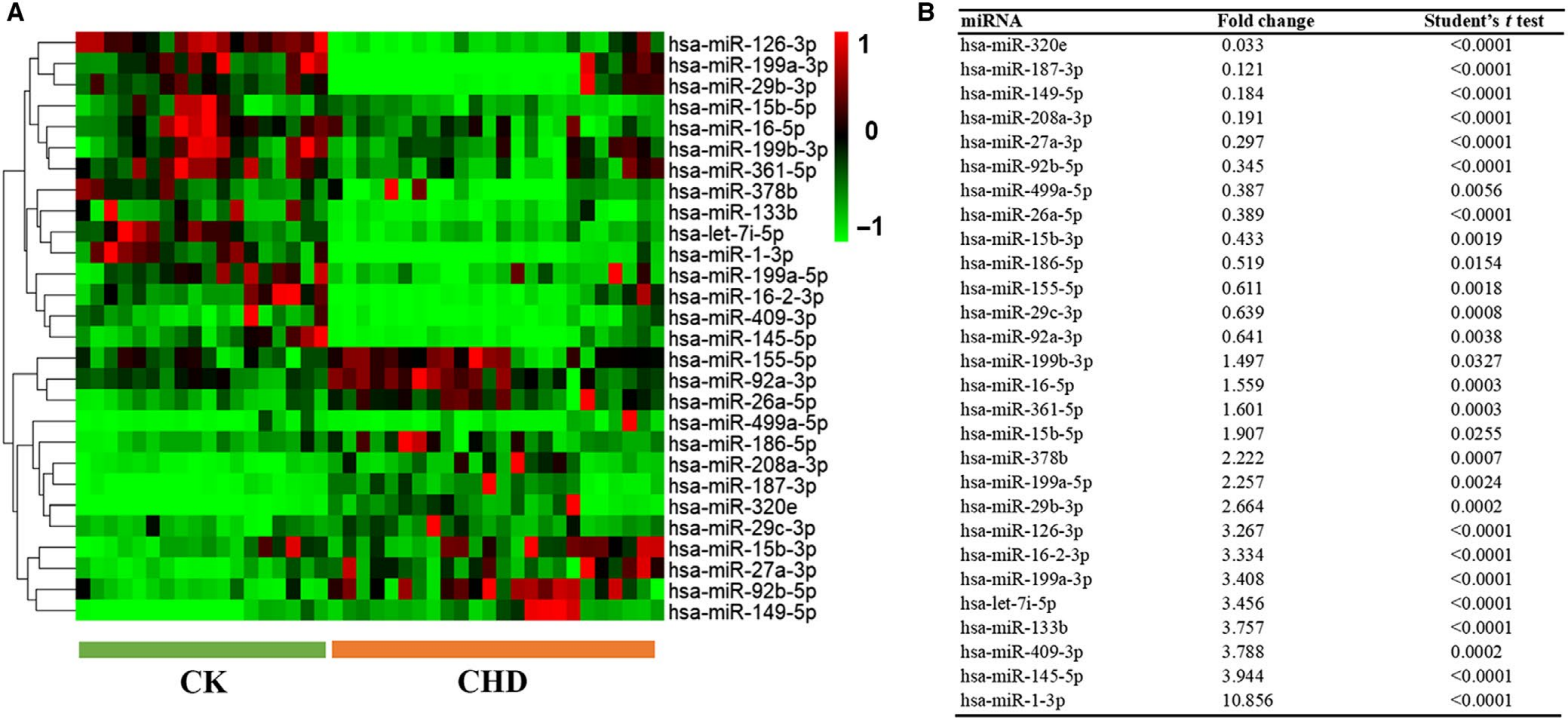
Cluster analysis of plasma miRNAs in training cohort. A, Heatmap showing the differentially expressed miRNA in 18 high‐risk controls compared to 24 CHD patients; (B) statistical results of differentially expressed miRNAs in plasma. The selection criteria are fold change >1.5 and *P* < .05. CHD, coronary heart disease

Because altered expression of larger numbers of miRNAs was found in CHD patients, we were increasingly intrigued to get an insight into altered miRNAs. To this end, we next employed S‐Poly(T)Plus based assay to confirm the expression of the candidate miRNAs selected from previous analyses. We arranged plasma into three sets including a training set and two verification sets. In training set, miRNAs were detected in a set of individual samples including 24 HCD patients and 18 high‐risk controls. Only those miRNAs with mean fold change >1.5 and *P* < .05 were chosen for further analysis. Moreover, miRNAs with Ct value >33 and detection rate <75% were excluded (Figure 3A). Based on these criteria, expression levels of 15 miRNAs including let‐7i‐5p, miR‐126‐3p, miR‐133b, miR‐1‐3p, miR‐145‐5p, miR‐15b‐5p, miR‐16‐2‐3p, miR‐16‐5p, miR‐199a‐3p, miR‐199a‐5p, miR‐199b‐3p, miR‐29b‐3p, miR‐378b, miR‐361‐5p and miR‐409‐3p markedly decreased in plasma from CHD patients, whereas expression levels of 13 miRNAs including miR‐149‐5p, miR‐155‐5p, miR‐15b‐3p, miR‐186‐5p, miR‐187‐3p, miR‐208a‐3p, miR‐26a‐5p, miR‐27a‐3p, miR‐29c‐3p, miR‐320e, miR‐499a‐5p, miR‐92a‐3p and miR‐92b‐5p significantly increased in CHD patients (Figure 3B; Figure S3).

### 3.4 Evaluation of miRNAs as sensitive and potential predictors for CHD in validation set‐1

**Figure 4:**
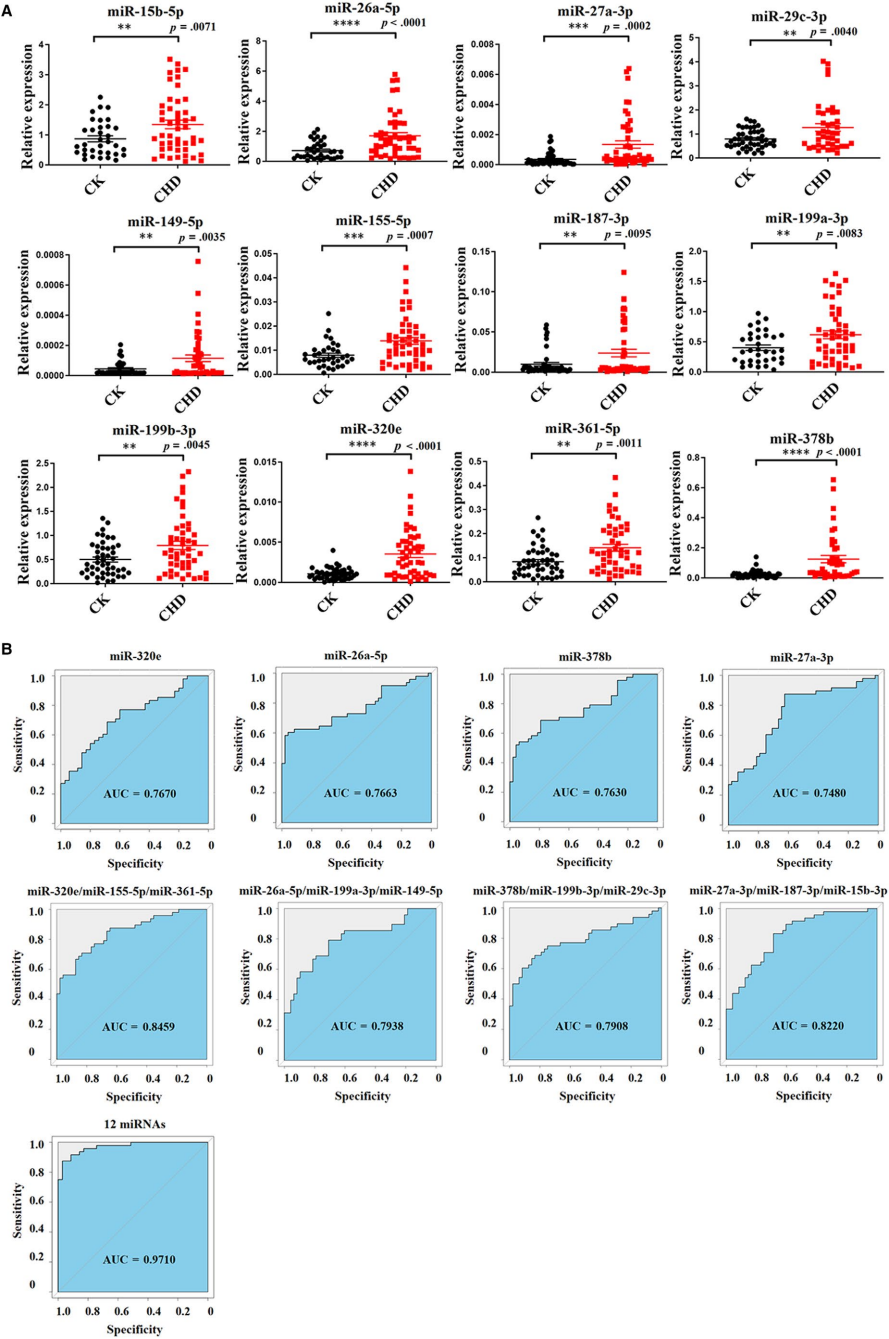
Candidate miRNAs were validated using CHD patients and high‐risk control individuals from validation set‐1. A, Plasma from 48 CHD patients and 48 high‐risk controls were detected with candidate miRNAs; (B) ROC analysis of individual miRNAs and combined miRNAs as biomarker for CHD diagnosis. Data are shown as mean ± SEM. CHD, coronary heart disease;CK, high risk control; ns, not significant, **P < .01, ***P < .001 and **** *P* < 0.001. *P* values are shown above each miRNA

The observation of significantly altered miRNAs in CHD and high‐risk controls inspired us to further validate them. Therefore, we validated candidate miRNAs with 48 CHD and 48 high‐risk controls’ samples randomly selected from validation cohort. According to the evaluation criteria which were identical with those in the training set, 12 miRNAs (miR‐15b‐5p, miR‐26a‐5p, miR‐27a‐3p, miR‐29c‐3p, miR‐149‐5p, miR‐155‐5p, miR‐187‐3p, miR‐199a‐3p, miR‐199b‐3p, miR‐320e, miR‐361‐5p and miR‐378b) were selected as potential biomarkers for CHD. miRNA quantitative analyses showed that the levels of these miRNAs were significantly increased in CHD patients (Figure 4A). To further explore the potential use of altered miRNAs as novel biomarkers for CHD, we built ROC (Receiver Operating Characteristic)curves and calculated the AUC (Area Under Curve)for these biomarkers, which ranged from 0.580 to 0.767, respectively (Figure 4B). To estimate the classification performance of the 12‐miRNAs‐based biomarker, we calculated the diagnostic sensitivity and specificity of this panel for CHD detection, which were 97.1% and 87.5%, respectively. Furthermore, the ROC curve for this panel revealed a pronounced diagnostic accuracy, evidenced by the AUC of 0.971 (*P* < .001), which was much better than that of 12 individual miRNAs (Figure 4B). These data suggested that these 12 circulating miRNAs might be a group of appropriate biomarkers for discriminating CHD patients from high‐risk controls.

### 3.5 Evaluation of miRNAs as sensitive and potential predictors for CHD in validation set‐2

**Figure 5:**
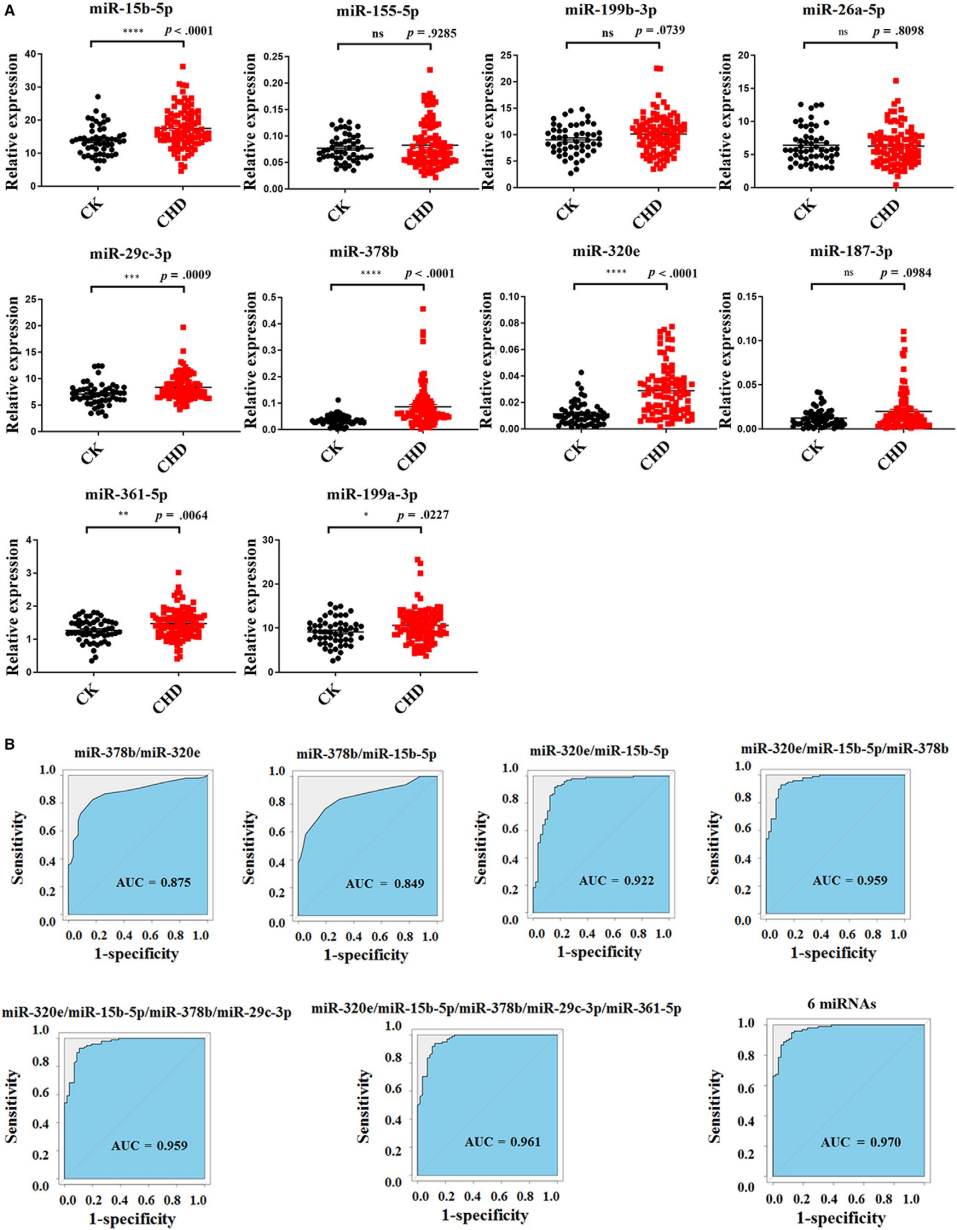
Candidate miRNAs were validated using CHD patients and high‐risk control individuals from validation set‐2. A, Plasma from 60 CHD patients and 95 high‐risk controls were detected with 10 candidate miRNAs; (B) diagnostic value of the combined miRNAs in CHD patients from second cohort. CHD, coronary heart disease; ns, not significant, * *P* < .05, ** *P* < .01, *** *P* < 0.01 and **** *P* < 0.001

After getting confirmation of twelve circulating miRNAs as novel biomarkers for CHD, we were sufficiently interested in investigating sensitivity and specificity of candidate miRNAs for CHD prediction. To this end, we assessed their levels using another independent validation set‐2 consisting of 95 CHD patients and 60 high‐risk controls. As is shown in Figure 5A, the expression alteration of six miRNAs (miR‐15b‐5p, miR‐29c‐3p, miR‐199a‐3p, miR‐320e, miR‐361‐5p and miR‐378b) was generally concordant between the validation set‐1 and 2, whereas there were no significant differences in the expression of miR‐26a‐5p, miR‐155‐5p, miR‐187‐3p and miR‐199b‐3p in CHD patients and high‐risk controls. Two miRNAs (miR‐27a‐3p and miR‐361‐5p) were excluded from the analysis with their detection rate <75%.

Moreover, we investigated the six miRNAs and their different combination panels in CHD cases and controls from validation set‐2. The individual miR‐320e, miR‐378b and miR‐15b‐5p could reliably discriminate CHD from controls with each AUC of 0.811 (95% confidence interval [CI] 0.602‐0.912), 0.784 (95% CI 0.592‐0.930) and 0.663 (95% CI 0.633‐0.702), respectively, whereas miR‐29c‐3p, miR‐361‐5p and miR‐199a‐3p showed a weaker performance with their AUC of 0.615 (95% CI 0.351‐0.867), 0.603 (95% CI 0.429‐0.832) and 0.581 (95% CI 0.418‐0.814) (Figure S4). Next, we combined the statistically significant miRNAs together as new biomarker which showed a better performance compared with individual miRNA (Figure 5B). The performance of the six miRNA combined panel for CHD detection in validation set‐2 was 92.9% and 89.5%, which indicated that this panel was really a comprehensive and specific indicator. We further evaluated the performance of these candidates in plasma, most of whose miRNAs alone could perfectly distinguish healthy volunteers from CHD cases, except miR‐26a‐5p with its AUC of 0.717 (95% CI 0.680‐0.990) (Figure S5). At the same time, a formula was estimated to predict the probability of having CHD based on the relative expression level of these candidates compared to spike‐in cel‐54 by performing the binary logistic regression analysis in SPSS. The relationship between the risk of having CHD and the relative expression of predictors in details is *p* = hsa‐miR‐15b‐5p + hsa‐miR‐320e × 552 + hsa‐miR‐378b × 182.

Taken together, these novel findings suggest that these six circulating miRNAs, especially miR‐15b‐5p, miR‐320e and miR‐378b could be used as sensitive and independent predictors for CHD.

### 3.6 The distribution of circulating miRNAs in plasma

**Figure 6:**
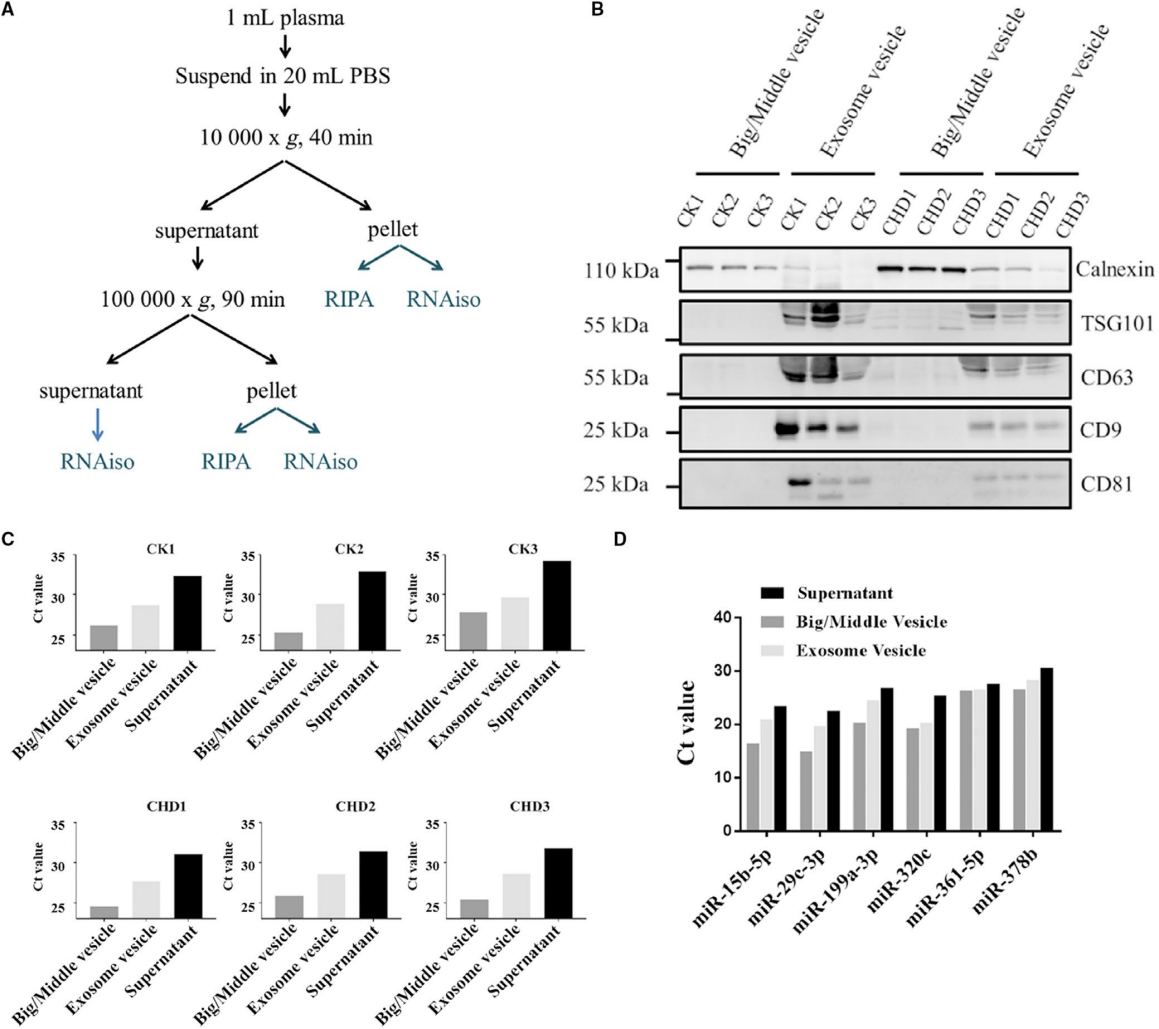
Exosomes isolation and quantification of miRNAs. A, Exosomes isolation flow chart is presented. B, Western blot characterization of exosomes by specific proteins of the exosomes and big EVs. C, The distribution of miR‐15b‐5p in different portion of plasma. D, Quantification of different candidate miRNAs from different portion of plasma. CHD, coronary heart disease; EV, extracellular vesicle

To investigate the distribution of these circulating miRNAs in plasma, we isolated EVs from plasma obtained from CHD patients and control individuals (Figure 6A). Exosome vesicle was confirmed by specific protein marker TSG101, CD63, CD9 and CD81, and meanwhile big/middle vesicles were detected by calnexin (Figure 6B). We analysed several miRNAs contents from different fractions of plasma, and our findings demonstrated that more than 80% miRNAs existed in the supernatant. About 15% miRNAs were assembled into big/middle vesicles and less than 5% miRNAs were packaged into exosome vesicles (Figure 6C,D). These observations indicate that free argonaut‐miRNA complex may be the main form of these circulating miRNAs existing in plasma, and only a small number of miRNAs are assembled into EVs.

### 3.7 Correlation of plasma circulating miRNA with angiographical and clinical factors

**Figure 7:**
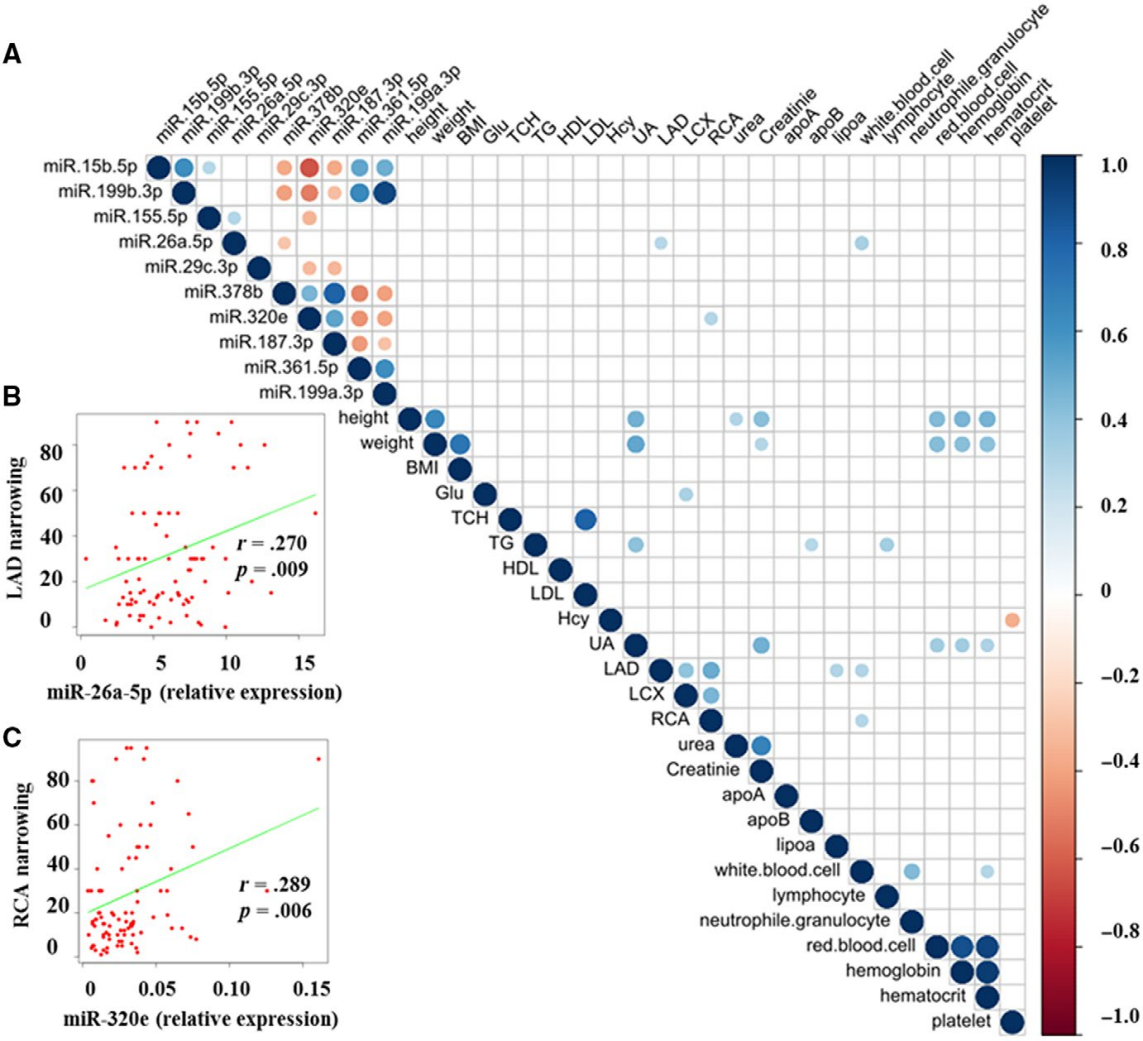
Correlation between miRNAs expression levels and clinical factors associated with angiographical results. A, Correlation of the miRNAs' expression levels with angiographical and clinical factors. Positive correlations are shown as blue dots; negative correlations are shown as red dots. Statistically insignificant correlations are excluded from analysis. B, Correlation between plasma miR‐26a‐5p and LAD luminal narrowing. Pearson correlation coefficient value and *P* value are shown in the figure. C, Correlation between plasma miR‐320e and RCA luminal narrowing. The scatter diagram demonstrates positive correlation between miR‐320e and RCA luminal narrowing. BMI, body mass index; Hcy, homocysteine; HDL, high‐density lipoprotein; LAD, left anterior descending; LCX, left circumflex branch; LDL, low‐density lipoprotein; RCA, right coronary artery; TCH, total cholesterol; TG, triglycerides; UA, uric acid

To determine whether the expression levels of these miRNA biomarkers are associated with clinical features of CHD patients, we estimated the correlation coefficient between miRNAs and angiographical/clinical factors (Figure 7A; Files S3 and S4). Our data revealed that the expression levels of miR‐26a‐5p and miR‐320e were significantly correlated with left anterior descending (LAD) and right coronary artery (RCA) luminal narrowing (*P* = .009 and .006, respectively) (Figure 7B,C), although the correlation coefficients were relative weak. High level of lipoprotein(a) (LPA) and large numbers of leucocytes were also correlated with LAD/RCA luminal narrowing (*P* = .004). Similar patterns of association were identified for glucose and left circumflex branch (LCX) luminal narrowing (*P* = .002). And the correlations between LAD and LCX and RCA narrowing were significant (*P* < .001) (Figure S6). These findings reinforce that circulating miR‐320e and miR‐26a‐5p may act as novel biomarkers for CHD diagnosis.

## 4 DISCUSSION

A growing body of evidence suggests that circulating miRNAs play a central role in identifying the occurrence and development of various diseases and may potentially serve as minimally invasive biomarkers.^22^ microRNAs are actively or passively released in the circulation, and prior studies have reported plenty of circulating miRNAs as promising biomarkers in cardiovascular disease^23^; however, the sensitivity and specificity of these biomarkers requires further enhancement. However, it has been a challenge to identify new miRNA biomarkers due to lack of innovative technology. From the patients' perspective, the improvement of the diagnostic situation should be urgent. Our work based on a direct quantification method that easily handles a large number of clinical samples (blood, plasma, serum and urine) is an important step forward as an auxiliary method for disease diagnosis.

Based on next‐generation sequencing and S‐poly(T) results,^19^, ^24^ we selected 343 mature miRNAs in plasma. By using the S‐Poly(T)Plus method (Figure 1B), we rapidly and accurately screened genome‐wide miRNAs in plasma from CHD patients and control individuals (Figure S2). The nominal EDTA concentration in blood samples is much lower than the concentration of MgCl2 in RT‐PCR and PCR reaction, so it has a slight effect on these reactions. Furthermore, previous study shows EDTA is a better anticoagulant than heparin and citrate for plasma preparation.^25^ In the present study, we ultimately selected a group of miRNAs as a first pass to introduce a specific and non‐invasive diagnostic tool for CHD. We propose that the expression pattern of all these miRNAs may make it possible to differentiate between high‐risk cases and CHD cases. Compared to high‐risk CK group, miR‐133b and miR‐1‐3p have lower expression in CHD patients while miR‐499 and miR‐208 have higher expression (Figure 3). All four miRNAs are muscle‐enriched, although miR‐499 and miR‐208 are usually expressed at extremely low levels except in cases of substantial (cardiac) muscle damage.^14^, ^26^, ^27^ Interestingly, our data showed that miR‐15b‐5p, miR‐29c‐3p, miR‐199a‐3p, miR‐320e, miR‐361‐5p and miR‐378b are dysregulated in CHD patients (Figures 4A and 5A), consistent with previous studies.^28^ Recent research has proved that miR‐15b‐5p serves as a target of *MALAT1*, which could active *mTOR* signalling pathway and affect cell proliferation, apoptosis and autophagy to mediate CAD progress.^29^ miR‐361‐5p, along with other miRNAs known to target *VEGF* directly, was dysregulated in CAD.^30^ A recent study demonstrates a significant up‐regulation of miR‐378 suggesting a novel endogenous repair mechanism activated in heart injury.^31^ More importantly, miR‐15b‐5p, miR‐320e and miR‐378b demonstrated superior performance in discriminating CHD cases from high‐risk cases. Moreover, the combination of these six miRNAs could distinguish CHD from control individuals at very high sensitivity (92.9%), specificity (89.5%) and AUC of 0.971 (Figure 5B). Furthermore, when we detected these miRNAs in healthy volunteers and CHD cases, these candidates adequately distinguished different types of plasma (Figure S5). Among the various miRNAs investigated in our study, miR‐15b‐5p, miR‐155‐5p, miR‐149‐5p, miR‐199a and miR‐378b^32^, ^33^, ^34^ have been reported to be correlated with CHD. Most importantly, for the first time our study showed that the increase of the expression level of miR‐361‐5p, miR‐29c‐3p and miR‐320e has a high correlation with CHD.

Previously, miRNAs have been reported to be transported in body fluids within exosomes, and once released into extracellular fluid, exosomes fuse with other cells and transfer their cargo to acceptor cell.^35^ Interestingly, our results showed that all the candidate miRNAs mainly existed outside of EVs (Figure 6C,D), which was consistent with the results of quantitative analysis of miRNA content of exosomes.^24^, ^36^ Correlation analysis indicated that miRNAs (miR‐26a‐5p and miR‐320e) could be better biomarkers for CHD diagnosis compared to most conventional clinical factors, such as apolipoprotein A (ApoA), apolipoprotein B (ApoB), LPA (Figure 7; Figure S6). Consistent with results of previous studies, immune system was involved in CHD patients,^37^ as leucocyte was correlated with RCA narrowing.

### 4.1 Study limitations

Because patients with myocardial damage were excluded from our cohort, we cannot detect different expression patterns of miR‐499 and miR‐208 in the following analyses. The weak correlations between miRNAs expression levels and luminal narrowing may be because of the quantification strategy of narrowing coronary artery, as single plaque stenosis in one coronary artery is hard to be distinguished from a diffuse stenotic disease in multiple vessels. As some of the participators were taking drug treatment which may cause differential expression of multiple miRNAs, the noise and difficulty of data analysis were increased.

## 5 CONCLUSION

In conclusion, our study of plasma circulating miRNAs showed a unique and reliable pattern of non‐invasive biomarkers that have the potential to be used for early diagnosis of CHD. The biological characteristics of CHD were better understood through the study, which was conducive to the exploration of new therapies for future clinical applications to improve therapeutic efficacy and pertinence of treatment.

## CONFLICT OF INTEREST

The authors declare that they have no conflict of interest.

## AUTHOR CONTRIBUTIONS

Conceptualization: Mingyang Su, Yanqin Niu, Deming Gou; data curation: Mingyang Su, Yanqin Niu, Quanjin Dang; formal analysis: Mingyang Su, Yanqin Niu; methodology: Mingyang Su, Yanqin Niu, Quanjin Dang; resources: Zhongren Tang, Daling Zhu; validation: Mingyang Su, Quanjin Dang; visualization: Mingyang Su; writing—original draft: Mingyang Su, Yanqin Niu, Deming Gou; writing—reviewing and editing: Mingyang Su, Yanqin Niu, Deming Gou.

## Data Availability

The data that support the findings of this study are available from the corresponding author upon reasonable request.
