# Supplementary figures and images for: Circulating microRNA profiles based on direct S‐Poly(T)Plus assay for detection of coronary heart disease
Source: J Cell Mol Med. 2020 Apr 28;24(11):5984–97. doi: 10.1111/jcmm.15001 (PMC7294166; doi:10.1111/jcmm.15001)

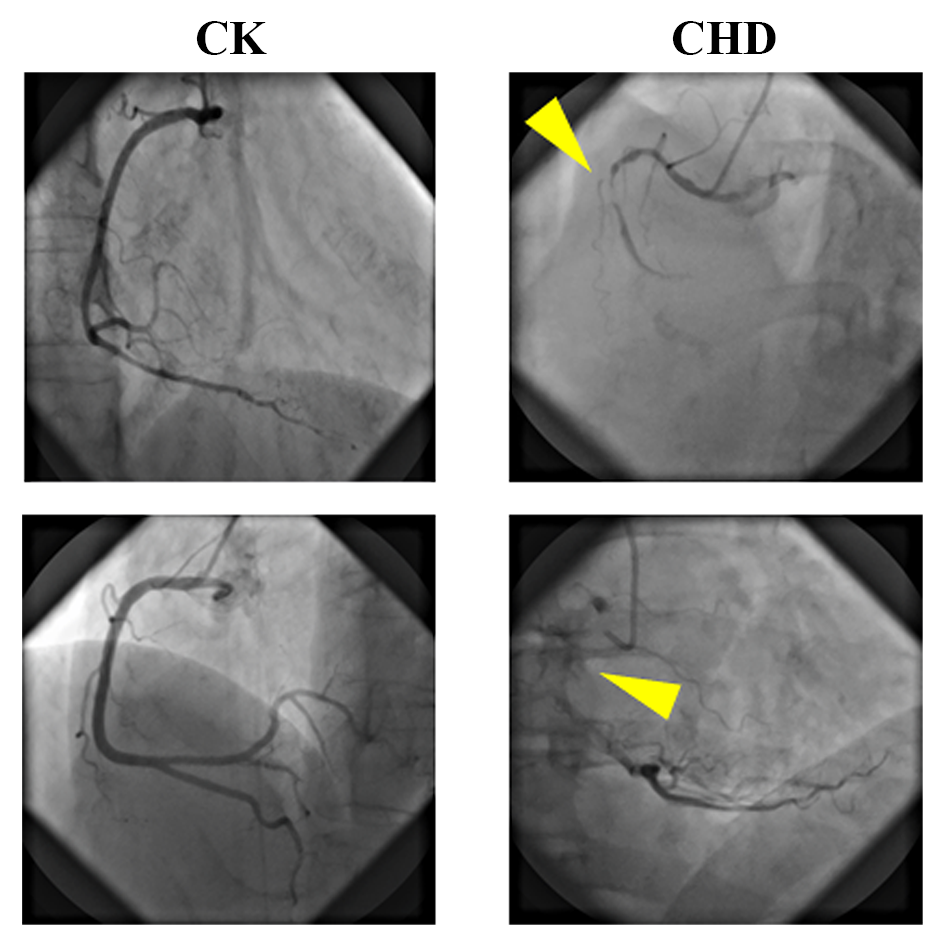

Supplement: Supplementary file 1 [file JCMM-24-5984-s001.tif]

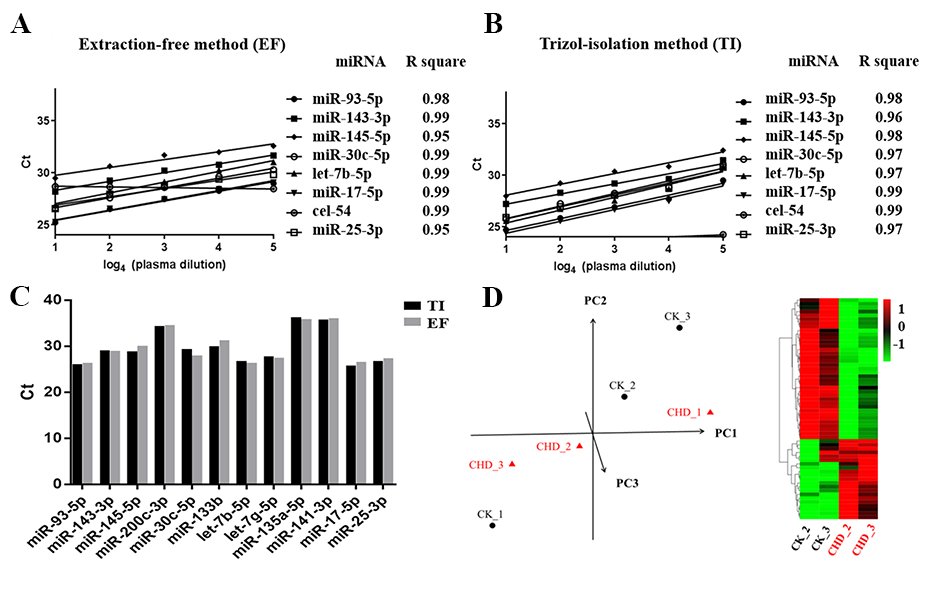

Supplement: Supplementary file 2 [file JCMM-24-5984-s002.tif]

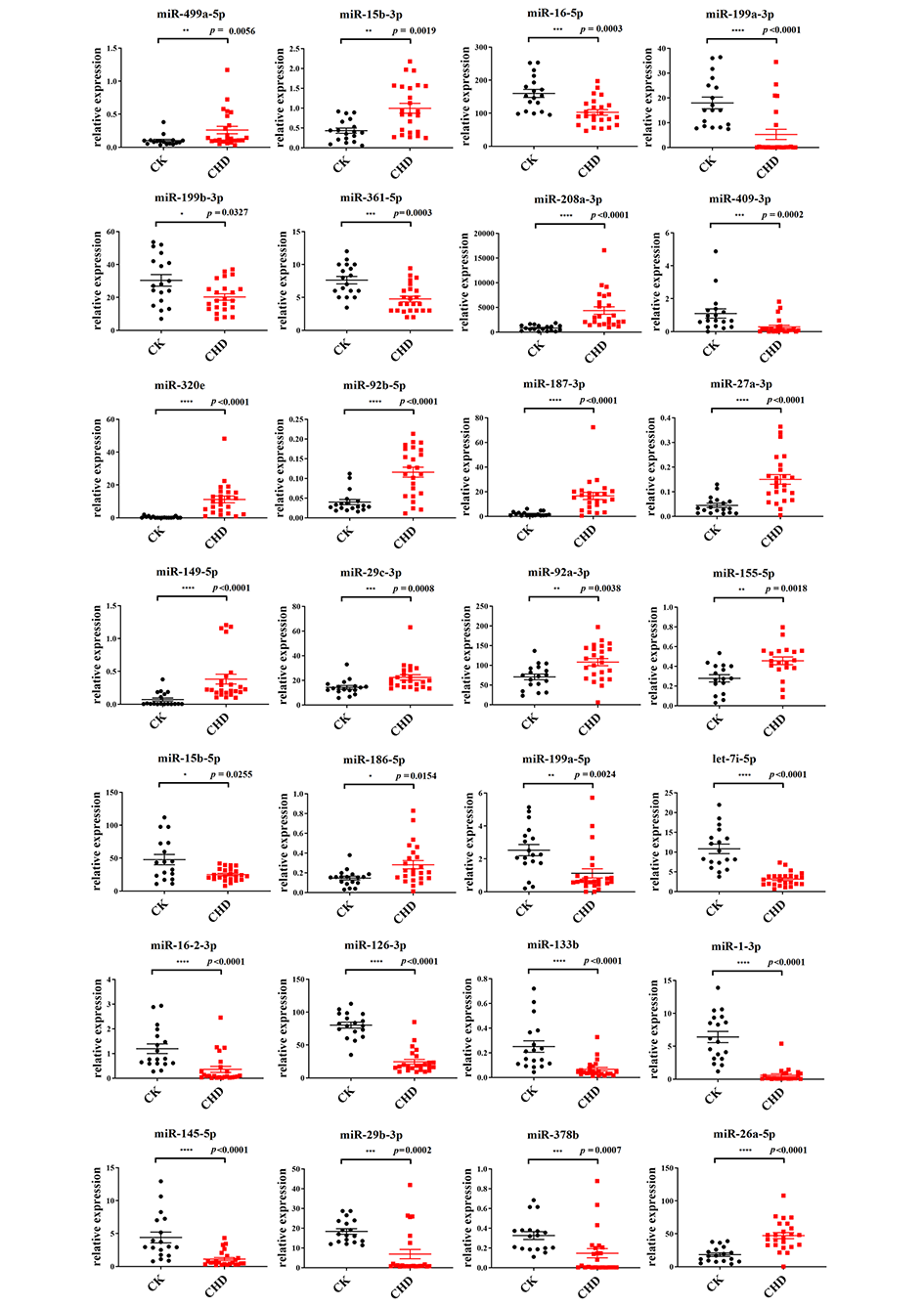

Supplement: Supplementary file 3 [file JCMM-24-5984-s003.tif]

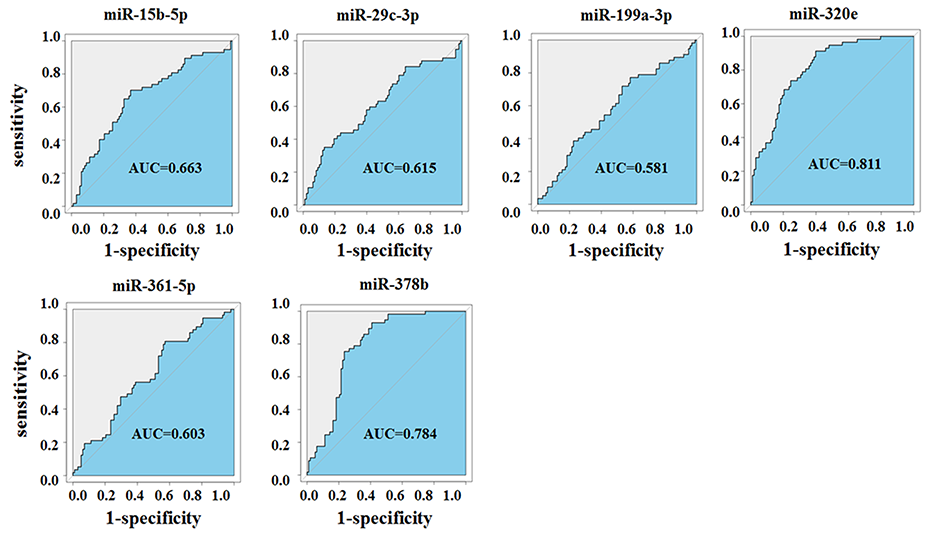

Supplement: Supplementary file 4 [file JCMM-24-5984-s004.tif]

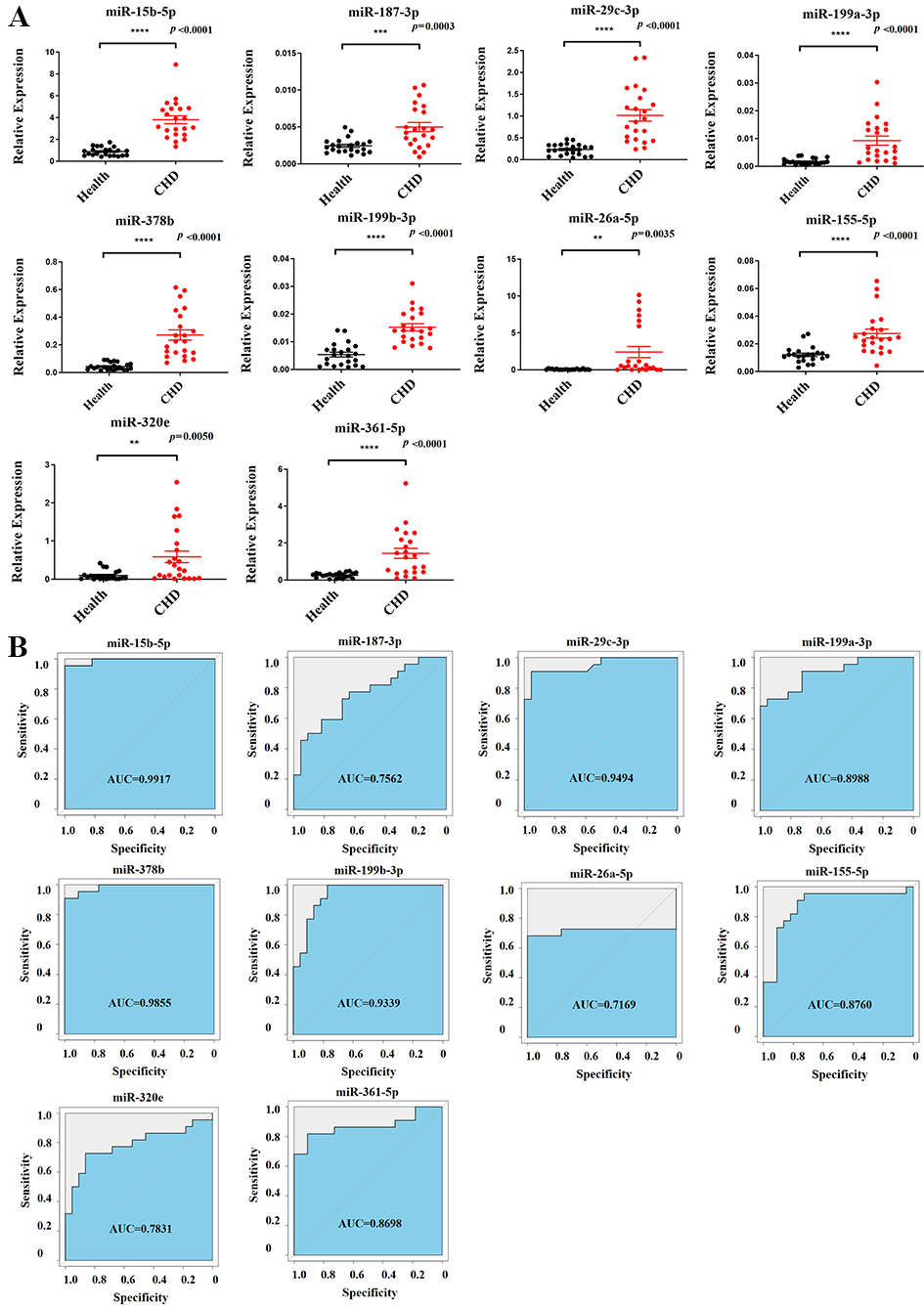

Supplement: Supplementary file 5 [file JCMM-24-5984-s005.tif]

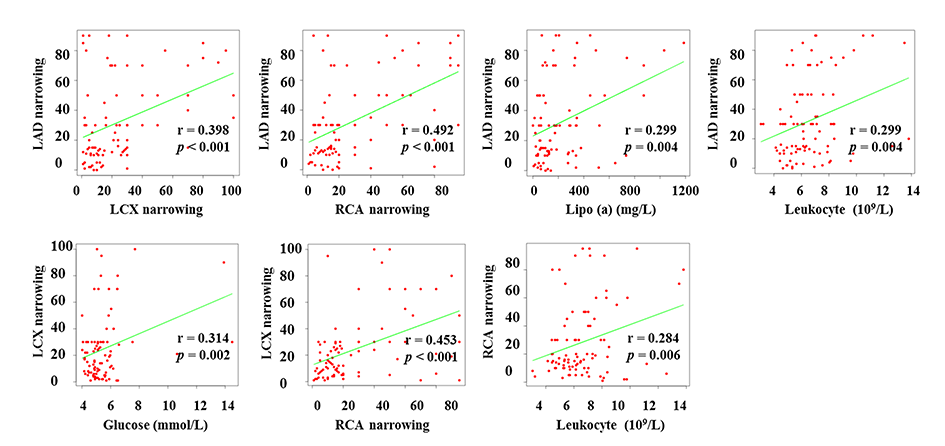

Supplement: Supplementary file 6 [file JCMM-24-5984-s006.tif]
