# Supplementary material for: Circulating microRNA profiles based on direct S‐Poly(T)Plus assay for detection of coronary heart disease
Source: J Cell Mol Med. 2020 Apr 28;24(11):5984–97. doi: 10.1111/jcmm.15001 (PMC7294166; doi:10.1111/jcmm.15001)
Supplement: Supplementary file 11 [file JCMM-24-5984-s011.docx]

**Figure S1 X-Ray angiographic image of high-risk controls and CHD patients.** High-risk controls’ angiographic images show clearly lumen of vessels. CHD patients’ images show narrowings or blockages inside the vessels.

**Figure S2 Methods comparison and Statistic analysis of pooled plasma.** (A-B) Dynamic range of extraction-free method (EF) and TRIzol-isolation method (TI) for miRNA quantification. (C) Sensitivity of the EF method and TI method. (D) PCA analysis shows that CK_1 and CHD_1 samples cannot be clustered with pooled high-risk plasma and pooled CHD samples; Heatmap shows the statistically altered miRNAs in plasma from pooled samples in discovery set.

**Figure S3 Statistic analysis of 28 candidate miRNAs in training set.** 28 candidate miRNAs are evaluated separately in CHD and high-risk cases. Results are shown as scatter plot. Data are shown as mean ± SEM. *p* values are shown above each miRNA.

**Figure S4 ROC curves of individual miRNA.**  The graph shows the ROC curve of these individual miRNA as biomarker in validation study.

**Figure S5 Candidate miRNAs were evaluated using CHD patients and healthy volunteers.** (A) Plasma from 18 CHD patients and 18 healthy volunteers are detected with 10 candidate miRNAs; (B) Diagnostic value of the 10 individual plasma miRNAs.

**Figure S6 Correlation between clinical factors and angiographical results.** Correlation between LAD luminal narrowing, LCX luminal narrowing, RCA luminal narrowing, high level of lipoprotein(a), and high number of leucocyte. Pearson correlation coefficient values and *p* values are shown in figures.**Supplementary material**

File S1. The sequences of mature miRNAs, specific F-primers, RT-primers, universal reverse primer and Taq-Man probe

File S2. The Ct values of circulating miRNAs in high-risk controls and CHD patients plasma measured by quick S-poly(T) method

File S3. Correlation coefficient result of statistical analysis of plasma circulating miRNA with angiographical and clinical factors

File S4. P-value of correlation analysis of plasma circulating miRNA with angiographical and clinical factors
